# Testing Two Online Symptom Checkers With Vulnerable Groups: Usability Study to Improve Cognitive Accessibility of eHealth Services

**DOI:** 10.2196/45275

**Published:** 2024-03-08

**Authors:** Kaisa Savolainen, Sari Kujala

**Affiliations:** 1 Department of Computer Science Aalto University School of Science Espoo Finland

**Keywords:** eHealth, online symptom checkers, usability, cognitive accessibility, web accessibility, qualitative research

## Abstract

**Background:**

The popularity of eHealth services has surged significantly, underscoring the importance of ensuring their usability and accessibility for users with diverse needs, characteristics, and capabilities. These services can pose cognitive demands, especially for individuals who are unwell, fatigued, or experiencing distress. Additionally, numerous potentially vulnerable groups, including older adults, are susceptible to digital exclusion and may encounter cognitive limitations related to perception, attention, memory, and language comprehension. Regrettably, many studies overlook the preferences and needs of user groups likely to encounter challenges associated with these cognitive aspects.

**Objective:**

This study primarily aims to gain a deeper understanding of cognitive accessibility in the practical context of eHealth services. Additionally, we aimed to identify the specific challenges that vulnerable groups encounter when using eHealth services and determine key considerations for testing these services with such groups.

**Methods:**

As a case study of eHealth services, we conducted qualitative usability testing on 2 online symptom checkers used in Finnish public primary care. A total of 13 participants from 3 distinct groups participated in the study: older adults, individuals with mild intellectual disabilities, and nonnative Finnish speakers. The primary research methods used were the thinking-aloud method, questionnaires, and semistructured interviews.

**Results:**

We found that potentially vulnerable groups encountered numerous issues with the tested services, with similar problems observed across all 3 groups. Specifically, clarity and the use of terminology posed significant challenges. The services overwhelmed users with excessive information and choices, while the terminology consisted of numerous complex medical terms that were difficult to understand. When conducting tests with vulnerable groups, it is crucial to carefully plan the sessions to avoid being overly lengthy, as these users often require more time to complete tasks. Additionally, testing with vulnerable groups proved to be quite efficient, with results likely to benefit a wider audience as well.

**Conclusions:**

Based on the findings of this study, it is evident that older adults, individuals with mild intellectual disability, and nonnative speakers may encounter cognitive challenges when using eHealth services, which can impede or slow down their use and make the services more difficult to navigate. In the worst-case scenario, these challenges may lead to errors in using the services. We recommend expanding the scope of testing to include a broader range of eHealth services with vulnerable groups, incorporating users with diverse characteristics and capabilities who are likely to encounter difficulties in cognitive accessibility.

## Introduction

### Background

Given the widespread use and popularity of eHealth services, there is a growing need for more accessible services to all potential user groups [1]. In recent years, more emphasis has been placed on accessibility and inclusion; for example, the European Union Accessibility Act has been incorporated into and enforced as national law since June 2022 [2]. As health care services are often public services, it is important that they serve a broad range of users. Furthermore, usability has been recognized as a key component of eHealth applications, and users may face problems with using the applications due to their health conditions [3]. In addition, patients with chronic illness have been reported to encounter more cognitive challenges [4]. Thus, extra attention should be paid to the usability of eHealth applications.

Universal design and design for all address these requirements by aiming at designing services that are usable by and accessible to all user groups regardless of their age, abilities, or possible disabilities [5]. Usability is a high-level term that indicates how a system can be used by specified users in a certain context of use to achieve specific goals with regard to effectiveness, efficiency, and satisfaction [6]. Accessibility, which is a part of usability, describes how a system can be used by people with the widest range of needs, characteristics, and capabilities [6,7]. Thus, accessibility covers all sorts of users with different limitations. A concept that has been addressed by several research papers [8,9] is web accessibility (or e-accessibility), which refers to the accessibility of web services.

**Figure 1:**
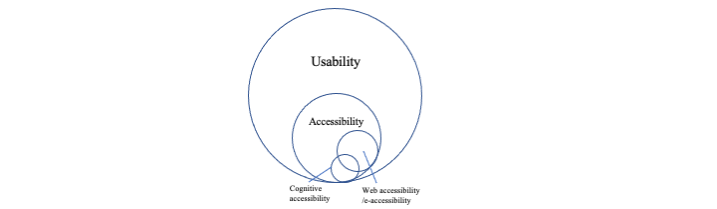
The relation of cognitive accessibility to usability and accessibility. Note that the sizes and positions of the circles are indicative.

In this paper, we address cognitive accessibility, which refers to accessibility beyond physical and sensory capabilities, and thus takes into account varied human characteristics such as intellectual disabilities, attention difficulties, reading problems, autism spectrum disorders, and low language skills [10]. Cognitive accessibility is an important aspect of web accessibility as it involves a large number of users and has a high impact on usability [10]. A summary of the relationship between these concepts is presented in Figure 1.

This research focuses on cognitive accessibility within the context of the 2 most frequently used online symptom checkers in Finnish public primary care across numerous municipalities in Finland. Online symptom checkers are used by people seeking health-related guidance, and these services typically provide an urgent assessment and suggest guidance based on the symptoms reported by the user [11]. Patients can use the 2 examined symptom checkers to book appointment times for doctors and laboratory tests or obtain medical help for the most common health issues. First, patients report their symptoms and submit them to the health care center through the symptom checker. Health care professionals receive patient inquiries with an urgency rating, decide on actions to be taken, and inform patients.

Patients are generally highly satisfied with symptom checkers, but younger and more highly educated people have been more likely to use them [11]. For example, symptom checkers enable patients to access health care anytime and anywhere. Therefore, it is essential to ensure that all user groups, including individuals in vulnerable situations, can use these services effectively. Symptom checkers can also empower users as a means of facilitating their health care [12]. However, the accuracy of the symptom checkers depends on how well patients are able to communicate their symptoms when using the tools [13]. As these services spread and are used by a wider range of individuals, it is crucial to also evaluate their usability and accessibility with a more diverse set of users.

### Prior Work

#### Vulnerable Groups

Many public eHealth services and their poor usability and accessibility can cause challenges for certain user groups [14]. These user groups are, thus, in a potentially vulnerable situation in using the service and at risk of digital exclusion [1]. This is especially problematic because research has shown that digital exclusion can cause social exclusion [15]. Public health services must, thus, address the needs of potentially vulnerable groups, including people who are disadvantaged by health, economic, cultural, or social conditions [16], such as older adults, migrants, mental health service users, and the unemployed [16,17].

Older adults are the largest group to face challenges in using digital health services [18,19]. As people age, their cognitive abilities may weaken, with cognitive load being identified as the most significant accessibility barrier for older adults [20]. Memory changes can also affect learning, information processing, and language comprehension [21,22]. Additionally, older adults often struggle with focusing their attention, particularly when multitasking [21,22]. Moreover, older age groups tend to use eHealth services less frequently than younger demographics. A Finnish study examining an online symptom checker (referred to as service A in this study) observed that individuals aged 20-39 years used the service more actively compared with older age groups, relative to their representation in the population [23]. This suggests that enhancing service usage entails prioritizing usability and accessibility from the perspective of older users as well.

Migrants represent a growing demographic that often faces challenges when accessing health services in their new country of residence [1]. Language barriers and a lack of digital skills are common issues encountered by this group [1]. Additionally, individuals with intellectual disability are another vulnerable population impacted by the digitalization of health services [24]. They have been noted to experience more difficulties in finding information on the internet and understanding online information compared with the general population [25].

Previous research suggests that vulnerable groups, such as older adults and individuals with mild intellectual disabilities, encounter cognitive challenges when using technology [26,27]. Therefore, the development of more accessible eHealth services would enable these groups to access health information more easily [25,28], thereby enhancing their sense of empowerment concerning their health issues.

The preferences or needs of older adults or individuals with mild intellectual disabilities are often overlooked in the majority of eHealth studies [29,30]. It is imperative to better consider these user groups during the design of eHealth services [17,28]. Many eHealth applications could greatly benefit from the application of universal design principles [29], which facilitate understanding the needs of potentially vulnerable groups and inform the design of more inclusive and usable services [31,32]. Consequently, this enables vulnerable groups to derive as much benefit from eHealth systems as the rest of the population [33,34]. Indeed, universal access approaches can offer benefits to anyone [35]. Therefore, to gain a better understanding of the challenges faced by vulnerable groups when using services, it is essential to conduct testing with a diverse group of users.

#### Usability Testing of Symptom Checkers

The usability of symptom checkers has been examined in prior research; however, there has been limited emphasis on potentially vulnerable user groups, such as older adults, migrants, and those with intellectual disability [36-38]. Moreover, research on usability in the eHealth domain frequently concentrates on quantitative aspects (eg, the number of errors, task completion times, and usability questionnaires) and typically involves a large number of users [12,36,38]. However, the qualitative aspect of usability studies is also crucial for gaining a deeper understanding of the thoughts and reasons behind errors, as well as capturing the patient’s perspective at a broader level [3,39]. Additionally, while a System Usability Scale (SUS) questionnaire provides a numeric score for experienced usability, it alone is not adequate for evaluating usability. Instead, it should be complemented with other measures, such as task completion rates or more qualitative approaches, to ascertain which aspects of a service require improvement and how best to address them [39].

Marco-Ruiz et al [13] conducted research on symptom checkers and emphasized the significance of testing with real users to comprehend the cognitive processes involved when using a new system to record health data. Furthermore, they noted that the user base accessing symptom checkers is highly diverse, with some individuals possessing higher health literacy and experience in recording online information, while others may have very limited or no experience [13].

### Goal of the Study

The goal of our study is to gain a deeper understanding of cognitive accessibility in the context of eHealth services. Therefore, our paper focuses on addressing the following research questions:

- What kind of challenges do vulnerable groups face in using eHealth services?
- What needs to be considered when testing with vulnerable groups?

The structure of this paper is as follows: In the next section, we describe the methods used in this study, followed by the presentation of results. Subsequently, we discuss the findings and overarching contributions of this study, concluding with our final remarks.

## Methods

### Approach and Researcher Background

Our qualitative study adopts a case study approach, wherein the cognitive accessibility of eHealth services was assessed through usability testing of 2 online symptom checkers. The research team comprised 3 researchers: The first researcher, a human-computer interaction student, conducted the initial 8 tests as part of their master’s thesis work. Subsequently, a second researcher, a senior researcher with expertise in human-computer interaction (who served as the thesis advisor), conducted the remaining 5 tests. Additionally, a third senior researcher with backgrounds in human-computer interaction and eHealth oversaw the entire study.

### Context and Study Setting

We conducted a usability test of 2 Finnish online symptom checkers in 2 phases in Finland during the Spring and Fall of 2021. The tested services were Omaolo (DigiFinland Oy) [40] and Klinik Access (Klinik Healthcare Solutions Oy) [41], which are the 2 most-used symptom checkers in Finnish public primary care. Omaolo has been actively used since 2019, while Klinik Access, which is also used internationally, has been in use since 2015. Both services are designed to assist patients in obtaining appropriate care. Users answer a set of questions regarding their symptoms, following which the symptom checkers use artificial intelligence to assess the urgency of care. If necessary, the services guide patients to contact emergency care services.

The Omaolo symptom checker comprises 15 specialized symptom checkers tailored for different types of symptoms, along with a generic symptom checker. Each symptom checker prompts the user with a specific set of questions and subsequently recommends the next steps they should take. Additionally, if the user provides their home municipality, the service displays recommended actions specific to the area, offers contact details, and may even facilitate direct contact with health care professionals if deemed necessary. The Omaolo symptom checker served as the primary COVID-19 symptom checker in Finland, enabling users to schedule appointments for COVID-19 tests. Consequently, its user base experienced a significant surge [23].

The Klinik Access symptom checker enables users to initially select the part of the body where their main symptoms are located. Subsequently, it prompts for more specific symptoms. The responses can then be forwarded to the medical staff responsible for the patient’s care before their appointment, ensuring the patient is directed to the appropriate type of health care professional. The primary distinction between these services lies in their user interface (UI): Klinik Access features a more visual UI with a list of clickable symptoms, whereas Omaolo presents users with multiple-choice questions describing the symptoms. Henceforth, the Omaolo service will be denoted as service A, and Klinik Access will be referred to as service B. It is important to note that both services are classified as medical devices and must adhere to specific safety requirements, such as repetitive questions, which may impact usability.

### Sampling Strategy

Purposive sampling [42] was used to recruit participants, who were sourced through personal contacts and various associations representing the targeted user groups. These associations included initiatives such as the Selkeästi meille, which focuses on enhancing cognitive accessibility, and Väylä ry, which is dedicated to improving the employment opportunities of individuals with intellectual disability. It is important to note that the test facilitator did not have a close personal relationship with the participants, such as being a friend or family member, during any of the test sessions.

A total of 13 participants were recruited to partake in the study. Notably, an evaluation of sample sizes within the field of human-computer interaction has indicated that 12 is the most common sample size for usability studies [43].

### Ethical Approval

The study received approval from the ethical review board of Aalto University (D/902/03.04/2021). Each participant provided informed consent by signing a consent form after confirming their understanding of the study’s purpose and how their information would be handled. Reporting has been conducted in such a manner that individual participants cannot be identified.

### Data Collection Methods

#### Overview

The main methods used in this study were thinking aloud, observations, questionnaires, and semistructured interviews. Before the actual tests, a pilot test was conducted to identify any potential inconsistencies and to ensure that the questions and instructions were comprehensible. Minor adjustments to the test setup were made based on the findings from the pilot test.

#### Test Procedure

**Figure 2:**
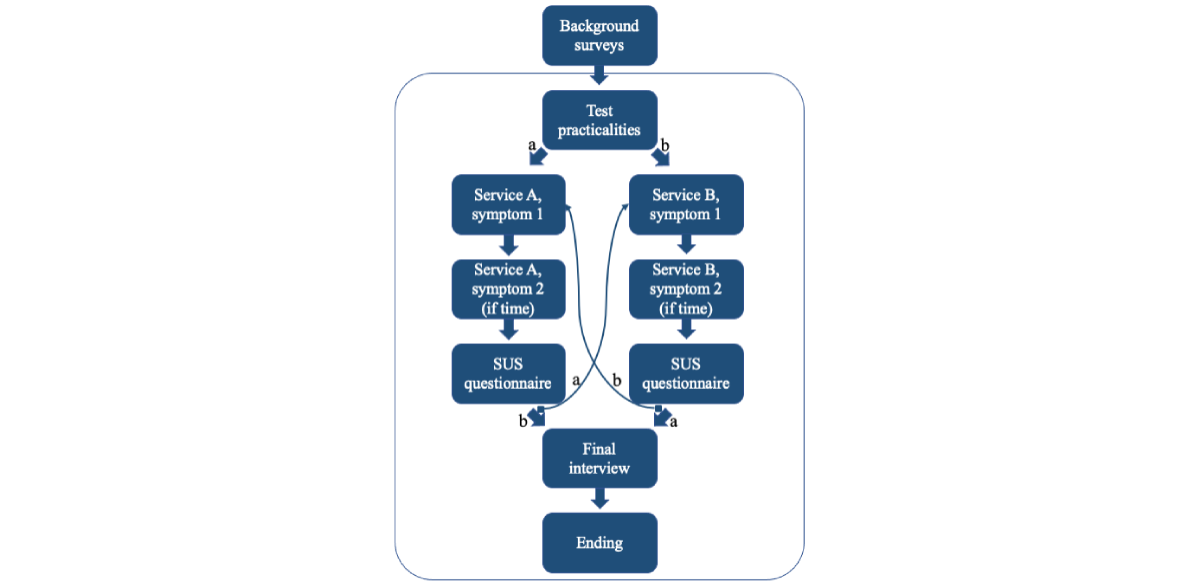
An overview of the usability test sessions with older adults, mildly intellectually disabled individuals, and nonnative speakers (N=13). Half of the participants started with Service A and the other half with Service B.

An overview of the test sessions is presented in Figure 2.

Each participant tested both services, and the order of service usage was counterbalanced. During the testing phase, participants were presented with 2 symptom vignettes, each providing a brief description of the symptoms they were instructed to imagine having. These vignettes were used 1 at a time. Participants were then asked to open the service and imagine they had the symptoms described in the first vignette, aiming to determine how they should proceed. The vignettes and mode of distribution between the participants are presented in Multimedia Appendix 1.

After using the first service, participants were instructed to take the second vignette and attempt to use the service again. However, if the first part of the test had exceeded 40 minutes, the second vignette was omitted for the first service to prevent the overall test time from exceeding 90 minutes. Following their interaction with each service, participants were asked to evaluate the respective service.

After testing the first service, participants were instructed to open the second service and follow the same procedure. Upon completion of both testing phases, participants were asked to compare the 2 systems and select the one they preferred.

### Data Collection Instruments

#### Test Sessions

The test sessions were conducted via the Microsoft Teams videoconferencing platform, which facilitates screen sharing, screen recording, and voice recording functionalities. The decision to conduct remote testing was primarily influenced by the COVID-19 pandemic situation, but it also aligned well with the nature of the tests, as the services being evaluated were online. Participants used their personal computers to access the services during the testing sessions.

#### Symptom Vignettes

To streamline the usability test and eliminate the necessity for participants to input their personal medical information into the services, each participant was provided with 2 standardized clinical vignettes featuring predefined symptoms. These vignettes were selected from a list compiled by Semigran et al [44], encompassing a total of 6 conditions with varying severity levels. The selection included conditions with different severity levels to account for the fact that individuals may use symptom checkers in both urgent and nonurgent situations [45].

In line with the recommendations provided by Semigran et al [44], the selected vignettes encompassed 3 categories of triage urgency: conditions necessitating emergency care, conditions warranting nonemergency care, and conditions deemed unnecessary for medical visits, thus manageable with self-care. Moreover, we opted for conditions commonly observed within the age group under study to ensure relevance. These conditions encompass ailments such as acute bronchitis, back pain, and meningitis. To ensure clarity and relevance to the participants, the selected conditions were translated from English to Finnish and simplified. The English versions of the vignettes used can be found in Multimedia Appendix 2.

#### Background Questionnaires

Before the actual test session, participants were requested to complete a brief background survey and the health literacy survey HLS-EU-Q16 [46]. The background information collected were the participant’s gender; age; the frequency of doctor visits in the preceding 2 years; the number of doctor-diagnosed medical conditions; their previous usage frequency of digital health care services; and their frequency of digital device usage, such as smartphones or computers. These questions aimed to ascertain whether participants met the study’s target demographic criteria in terms of age and their ability to independently use electronic devices such as computers. The health literacy survey provided insights into participants’ understanding of health-related topics.

#### Interview and Questionnaire

After interacting with each service, participants were asked to evaluate the tested services. This involved administering an SUS questionnaire [47] to gauge the perceived usability of the system, as well as posing 4 interview questions:

- Would you use the service again in the future?
- Were the summary and the instructions about what to do next clear enough?
- Would you actually follow the instructions given?
- Given the option, would you use the service using your phone?

### Data Processing and Analysis

The test sessions were recorded using Microsoft Teams. The voice recordings of the initial 8 tests were transcribed in full, while for the remaining 5 tests, notes were taken from the recordings, and user comments were documented to streamline the process. An experienced researcher could identify the issues encountered by users as well as their comments without requiring a complete transcription. The notes and transcriptions underwent anonymization. Qualitative content analysis was used in this study. Using the notes and recordings, all usability issues were identified and compiled. This encompassed problems mentioned by participants as well as those observed during testing or evident from the recordings. The identified usability problems were coded and categorized based on their similarities. When new problems were identified, they were compared with existing ones, and if deemed similar, they were grouped under the same code. Eventually, these groups were consolidated under higher-level descriptive categories. Furthermore, user comments were collected to bolster the analysis and reporting process.

The background questionnaires were analyzed by aggregating the responses to obtain an overview of participant characteristics. Additionally, the health literacy surveys were analyzed according to the guidelines [46] to determine the groups to which participants belonged. The SUS questionnaires were analyzed by computing the SUS scores as per the guidelines [47], resulting in scores of up to 100 points, which were then compared with the general score.

To ensure the quality and trustworthiness of the study, a senior researcher (the second author) supervised the entire research process and provided support for the analysis work. Two other researchers (the first author and the master’s thesis worker) conducted the actual tests and analyzed the data. Therefore, a total of 3 researchers participated in the process, ensuring that data gathering and processing proceeded appropriately.

## Results

### Overview

The subsequent sections present the principal findings of the study. We commence with an overview of the participants’ characteristics, followed by an examination of the identified usability issues. Finally, we present additional findings that emphasize the characteristics of these user groups.

### Test Participants

A total of 13 individuals participated in the study in Finland. Among them, 4 were individuals with mild intellectual disability, 4 were older adults (aged 75-79 years), and 5 were nonnative Finnish speakers. Therefore, all test users potentially encountered cognitive accessibility challenges with the services. The background characteristics of the participants are detailed in Multimedia Appendix 3.

**Figure 3:**
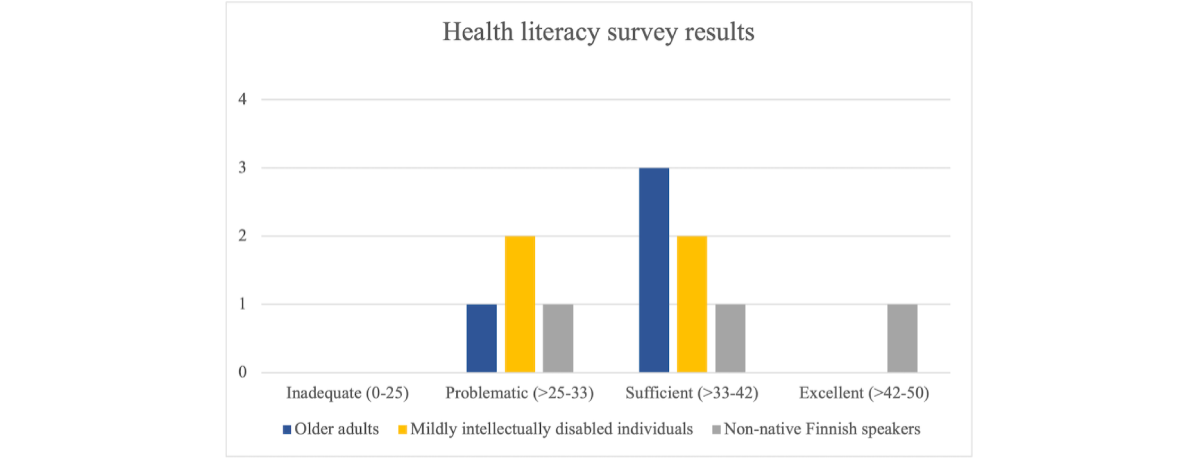
The results of the HLS-EU-Q16 health literacy assessment of older people, mildly intellectually disabled individuals, and non-native speakers divided into four categories.

The HLS-EU-Q16 questionnaire results were calculated in accordance with the guidelines [46], with each participant receiving a score corresponding to a quartile representing their health literacy level. The results were computed only for participants who responded to at least 80% of the questions, as recommended in the guidelines [46]. The questionnaire includes an “I don’t know” answer option, which was interpreted as the question not being answered. Consequently, the results of 2 of the nonnative participants were excluded, as they chose this answering option too frequently. Figure 3 depicts the distribution of health literacy among the 3 groups.

### Usability Problems

#### Cognitive Accessibility Issues

The study identified a total of 65 usability problems with the 2 systems. Specifically, 36 usability problems were discovered with service A, while 29 problems were identified with service B. These issues occurred across 99 and 91 individual user instances, respectively. The problems were classified into 14 usability problem categories. A comprehensive list of the usability problem categories is provided in Multimedia Appendix 4. For the purpose of this discussion, we will focus on issues related to cognitive accessibility, primarily concerning terminology, text volume, and UI clarity.

#### Terminology-Related Issues

The most prevalent issues were associated with terminology and answering options. eHealth services frequently incorporate specialized language and specific terminology, posing challenges for users with cognitive limitations. Nearly all users encountered confusion with certain terms or inadvertently mixed them up with similar ones. Furthermore, lengthy words and extensive blocks of text, such as lengthy paragraphs, presented challenges, a sentiment that was also echoed during the interviews. Users with cognitive restrictions often encounter challenges when confronted with long words and extensive passages of text.

As one user commented,

It takes time to go through all the texts.

ID10, nonnative

Related issues were reported and commented on by users across all user groups. In addition to contributing to usability problems, these issues slowed down the usage of the services and occasionally led users to select incorrect symptoms.

#### Issues Related to the Clarity of the UI

Another area where users encountered difficulties was with the visibility of information and the lack of clarity in the UI. It is crucial for the most important information and elements of the UI to be clearly visible, facilitating easy comprehension for users. Additionally, problems arose when users’ attention was diverted to unimportant features. These issues are especially pronounced among user groups with cognitive difficulties, as they require additional attention to comprehend the content and must focus more intently. Furthermore, some users found the input methods challenging; initially, they struggled to discern the type of information required for input in a field and how the inputting should be performed.

The most prevalent individual usability problems we identified regarding the logic and functionality of the UI, observed across all 3 user groups, are detailed in Textbox 1.

**Individual usability problems identified.**

1. **Users making an incorrect selection due to an item being highlighted in the user interface:**
  Service A was, at the time of the study, the prevalent symptom checker for COVID-19 in Finland; COVID-19 was highlighted at the top of the home page of service A and was thus the first item to attract the users’ attention and be selected.
2. **Difficulties in making the correct selection from a long list of items:**
  Service A had a list of 15 symptom checkers from which the user had to choose, making it difficult for the users to select the correct symptom checker to continue with.
3. **Not remembering what questions needed to be answered after the questions disappeared:**
  Service B presented questions as placeholders to describe symptoms in open answers, and these questions disappeared when the user started typing in the field; as a result, the user might not fully describe their symptoms.
4. **Being confused by long lists of apparently uncategorized symptoms:**
  Service B had long lists of symptoms as selectable buttons that seemed to be unorganized and caused anxiety and confusion.
5. **The logic and functionality of submenus were not understood by the users**:
  Service B had additional submenus and dialog boxes that were not fully understood by the users. There was a small arrow that opened the submenu and the logic of how the items were selected or the submenus opened was unclear.
6. **The users did not understand the logic of the input fields that combined several user interface items:**
  The way in which service B required the duration of symptoms to be input meant that the user needed to enter the number in one field and then select the unit from different options. However, the unit selection was not clearly related to the textbox where the user inputs the number.

These individual usability problems highlight issues with how information is presented to users, with clarity being particularly emphasized among this user group. In some instances, the selection or input options were unclear, and the services featured lengthy lists of symptoms.

Clarity was a recurring theme in several test sessions. As one user commented:

...if you think about this in real life, if you have a fever and you’re doing this and you start to scroll all these selection choices and you’re evaluating which one would fit best, the options are quite broad, so it might be quite difficult to do in practice...

ID7, user with mild intellectual disability

Similarly, one user suggested:

I don’t know you could kind of put those in order like one row and another row, these are quite...your eye kind of jumps, but otherwise those are clear.

ID2, older adult

One user preferred the structure of service A and, again, referred to the clarity with which the information is presented:

Well, [I prefer Service A] because it was maybe better organized, there was one thing and one question and then one answer. After this, the next question and so on. In the other one [Service B], you had to read all the small boxes and look for your symptom. [...]

ID8, user with mild intellectual disability

Well, maybe what is the most [difficult], this one had so many small boxes that at least for me, it was difficult to find my own symptom, the one I needed to select from there. So, if I wanted to know what fit me, I had to read through them all and then, since they are not in any order, they just are there, I had to read them all, to see if I could find the one I have at the moment.

ID8, user with mild intellectual disability

It is worth noting that the symptoms were arranged in alphabetical order; however, the layout was such that users did not realize this ordering method had been used.

### Differences Between the User Groups

Some differences between the user groups were evident, although the majority of the usability problems were consistent across all user groups. Nonnative Finnish speakers found the service to be particularly slow to use, often taking an extended period to read the texts. One user commented regarding service B that:

Reading and writing text is not easy for an immigrant. When you can click on an item it is easy, you don’t have to write.

ID13, nonnative

The older adults did not encounter as many issues with longer texts. Instead, they faced more challenges in understanding the logic of the services and remembering to scroll down to view all the provided information. However, this scrolling also frustrated some nonnative users; as one user commented:

And again, we’re scrolling, this is terrible!

ID12, nonnative

The task completion times were also measured and presented for the initial tasks of both services. As depicted in Table 1, aside from the older adults, there were no significant differences in the completion times between the services. However, for the older adults, service B, which featured more clickable elements to choose from, appeared to be quicker to use. Nonnative speakers took the longest time to complete the tasks, primarily because they often needed to translate some of the terms used in the services. Three of the users used an online translator (eg, Google Translator), and at times, users asked the facilitator about specific terms. Overall, the task completion times were quite lengthy, suggesting that these user groups require ample time to use these services effectively.

**Table 1:** Average task completion times (first task) for both services. For older adults there was a clear difference in favor of service B; for the 2 other groups service A got a slightly better time.

Task completion times	Service A, hh:mm:ss^a^	Service B, hh:mm:ss
Older adults	0:14:30	0:08:00
User with mild intellectual disability	0:14:54	0:16:00
Nonnatives	0:15:46	0:18:16

^a^hh:mm:ss: hours:minutes:seconds.

For the few users who had the opportunity to test the services twice, the second time was generally much faster than the first, indicating good learnability. As one user mentioned:

Now I know that I need to select this and not the other, which I didn’t know previously.

ID8, user with mild intellectual disability

The SUS scores are provided in Multimedia Appendix 5, illustrating how participants evaluated the usability of the services. The SUS score ranges from 0 to 100 points. It has been assessed for numerous services, and according to Bangor et al [48], a satisfactory SUS score is above 70, with superior products typically scoring 80 or higher. However, it is important to note that the interpretation of SUS scores can vary depending on the type of product and its development phase. When evaluating the SUS scores of the tested services, which are predominantly below 75, it is evident that the perceived usability was not considered very good, except for nonnative Finnish speakers, as their scores hovered around 80.

From the interviews, we found that older adults tended to prefer computers over mobile devices when using the symptom checkers, whereas nonnative speakers mostly preferred mobile devices. The preference among users with mild intellectual disability was evenly divided. Nonetheless, the advantage of this type of online symptom checker was evident, as all participants expressed willingness to use the services again. The nonnative participants particularly valued a service that enabled them to input information at their own pace, as opposed to speaking on the phone. However, their preference for the service they would use was fairly evenly split, with no clear consensus: 7 participants favored service A, while 6 participants favored service B.

## Discussion

### Principal Findings

Testing for cognitive accessibility with 2 symptom checkers revealed that older adults, individuals with mild intellectual disability, and nonnative speakers may encounter numerous challenges when using the services. Primarily, problems arise concerning the terminology used. This highlights the need for greater emphasis on ensuring that the vocabulary used in the health sector, while specialized, remains understandable to a broad audience when services are intended for universal use. Furthermore, complications arose from the intricate structure and layout of the services. The significance of simplifying services, minimizing lengthy lists, and using more understandable terminology was highlighted in nearly all the test sessions. Implementing these improvements to the services would likely benefit a broader range of users [5].

There were distinct differences observed among the 3 user groups. Primarily, nonnative speakers assigned notably higher usability ratings to the services compared with the other 2 groups. One possible reason for this could be their overall satisfaction with the existence of such services, which enable them to seek help for their health issues without having to converse over the phone in a language that is not their native tongue.

One notable distinction between the user groups pertained to their preference for using either a computer or a mobile device. It was evident that older adults favored using computers, likely because of their larger screens and the familiarity that older adults have with them. Conversely, most nonnative Finnish speakers showed a preference for mobile devices, with some noting that they solely rely on their mobile devices and do not even own a computer. This preference may be influenced in part by financial constraints, which limit the number of devices a person can afford. Additionally, in our sample, older adults encountered fewer difficulties with processing long pieces of text compared with the other groups.

The promotion of online symptom checkers as a means to decrease unnecessary clinic visits [13] underscores the importance of ensuring they do not inadvertently increase contact with health care staff. Therefore, greater attention should be directed toward enhancing the cognitive accessibility of these tools, thereby enabling a wider range of users to use them effectively. In this study, users’ incomplete understanding of the questions or answer options led them to select additional symptoms, resulting in more serious care recommendations and advising users to seek emergency health care.

In ensuring the cognitive accessibility of eHealth services, it is imperative to involve vulnerable groups in testing. Testing with vulnerable groups provides valuable insights. First, it emphasizes the need for well-planned test sessions with a manageable number of tasks. This approach ensures that participants can fully engage and provide meaningful feedback without being overwhelmed. All of these groups required considerable time to complete the test tasks, with most participants unable to finish both planned tasks with either service. Moreover, they necessitated more detailed instructions and support during the test sessions, as many participants within these groups were not at ease with using eHealth services.

Based on the findings of this study and as supported by the broader universal design literature [5], several design guidelines can be outlined. Foremost among these is the emphasis on clarity. (1) The options provided to the user should be clear and understandable. The user should understand what the differences between different options are and what actions are available for them. (2) It should be made clear to the user where they should be focusing on. This is particularly important in services that contain a lot of information and options. (3) Long or uncommon words and difficult compound words should be avoided. This is especially relevant in health-related terminology, as the user might not understand the special terms and might confuse different terms. (4) Navigating the services should be easy and effortless. The user should be presented with as few options as possible, and excessive scrolling should be minimized. This is because the user may inadvertently overlook relevant information.

### Limitations

There are, naturally, some limitations to this study. First, the sample size of 13 participants was rather small, albeit quite typical for this type of qualitative study [43]. However, given the diverse nature of the user group and potential challenges related to cognitive accessibility, a more diverse participant pool could have been beneficial. Specifically, a wider age range of older adults could have been tested, considering their versatility as a group. Additionally, nonnative Finnish speakers could have been recruited from a more geographically diverse range of countries of origin. Moreover, testing should involve other diverse human characteristics, such as neurodiversity (including conditions such as attention-deficit/hyperactivity disorder, attention-deficit disorder, and various forms of autism). Given society’s rapid transition toward digitalized services, it is crucial to broaden the scope to include other groups at risk of digital exclusion.

Another limitation of this study is its focus on only 2 online symptom checkers. While the range of available online symptom checkers is already extensive, it is important to include testing of other eHealth services designed for use by all citizens. Additionally, this study only examines a limited list of symptoms and assesses usage on a 1-time or 2-time basis.

In conclusion, we recommend conducting testing with a more diverse user group, with a specific focus on accessibility and cognitive accessibility. Additionally, adopting a broader test setup that encompasses a wider range of symptoms and includes other eHealth services intended for broad usage would be beneficial.

### Comparison With Prior Work

Usability issues were efficiently identified during testing with special user groups. In a study by Liu et al [36], which involved 350 participants, similar problems were discovered with service A as found in our study. The authors observed comparable challenges related to understanding questions and terminology, along with a need to enhance the visual layout and instructions for users. However, a notable disparity was observed in completion times: their participants completed the symptom checkers in an average of 4 minutes and 9 seconds, whereas users in our study required, on average, 3 times longer. In addition to uncovering issues that notably impact cognitive accessibility, our study identified similar usability problems as other assessments. Furthermore, as highlighted by Jormanainen et al [23], the same service was used over 1.5 million times for COVID-19 evaluation, suggesting its successful use by a vast number of users. Moreover, challenges with terminology have been recognized in other services [20].

This study has concentrated on cognitive accessibility with 3 distinct user groups. Comparable user groups have been used in other studies that center on eHealth services [1,29,30]. Upon comparing our findings with these studies, we observe that the necessity for clearer language and terminology, along with the clarity of the service, has previously been recognized through interviews and focus groups [1,29]. Our study provides more nuanced insights into how these issues manifest in practical usage.

### Conclusions

In this study, we conducted a qualitative usability evaluation of 2 online symptom checkers, with a particular emphasis on the cognitive accessibility of the services. The evaluation targeted potentially vulnerable groups at risk of digital exclusion. Three distinct user groups participated in the tests: older adults, individuals with mild intellectual disabilities, and nonnative Finnish speakers. Our findings revealed that these groups encountered numerous difficulties with the tested services, particularly concerning their clarity and the language/terminology used. Furthermore, when testing with these groups, several key points must be considered: test sessions should be meticulously planned, instructions need to be clear, sessions should not be overly prolonged, and sufficient time must be allocated for each task.

In general, we found that testing with vulnerable groups was both useful and efficient. The rate of usability problems identified was notably high compared with the number of participants, and these issues were readily uncovered. These user groups encountered similar challenges related to information processing. It is imperative to provide them with better support through services that are clear, presenting less information and fewer options at once, and incorporating fewer long and complex words and selection lists. Additionally, following the principles of universal design, the proposed improvements are such that they will also benefit a more general user group. Therefore, we highly recommend testing with potentially vulnerable groups and, furthermore, expanding the user groups to include a representation of a broader variety of cognitive characteristics and challenges.

## Appendix

Multimedia Appendix 1 The distribution of the symptoms and services.

Multimedia Appendix 2 Symptom vignettes used.

Multimedia Appendix 3 The background information of the participants. All the participants used digital services multiple times a day. MID: mildly intellectually disabled.

Multimedia Appendix 4 Usability problem categories.

Multimedia Appendix 5 Average SUS scores for both services. For older adults the two services got the same results, for the two other groups Service A got a slightly better score.

## Abbreviations

SUS: System Usability Scale
UI: user interface

## Data Availability

The data sets generated during or analyzed during this study are not publicly available due to the sensitive nature of the data but the numeric data are available from the corresponding author on reasonable request.
