# Supplementary material for: Testing Two Online Symptom Checkers With Vulnerable Groups: Usability Study to Improve Cognitive Accessibility of eHealth Services
Source: JMIR Hum Factors. 2024 Mar 8;11:e45275. doi: 10.2196/45275 (PMC10960212; doi:10.2196/45275)
Supplement: Multimedia Appendix 1 [file humanfactors_v11i1e45275_app1.docx]

## **Multimedia Appendix 1 – the Distribution of the Symptoms and Services**

| Sub-group | Symptom 1 | Symptom 2 | First service | Second service |
| --- | --- | --- | --- | --- |
|  |  |  |  |  |
| Older adults | Back pain | Pneumonia | Service B | Service A |
| Older adults | Pneumonia | Back pain | Service A | Service B |
| Older adults | Deep vein thrombosis | Back pain | Service A | Service B |
| Older adults | Back pain | Deep vein thrombosis | Service B | Service A |
| MID | Influenza | Meningitis | Service A | Service B |
| MID | Acute bronchitis | Back pain | Service B | Service A |
| MID | Meningitis | Influenza | Service B | Service A |
| MID | Back pain | Acute bronchitis | Service A | Service B |
| Non-native | Influenza | Meningitis | Service A | Service B |
| Non-native | Acute bronchitis | Back pain | Service B | Service A |
| Non-native | Meningitis | Influenza | Service B | Service A |
| Non-native | Back pain | Acute bronchitis | Service A | Service B |
| Non-native | Back pain | Pneumonia | Service B | Service A |
