# Supplementary material for: Testing Two Online Symptom Checkers With Vulnerable Groups: Usability Study to Improve Cognitive Accessibility of eHealth Services
Source: JMIR Hum Factors. 2024 Mar 8;11:e45275. doi: 10.2196/45275 (PMC10960212; doi:10.2196/45275)
Supplement: Multimedia Appendix 2 [file humanfactors_v11i1e45275_app2.docx]

## **Multimedia Appendix 2 – Symptom Vignettes**

*Requires emergent care*

Meningitis – Imagine your head has been hurting and you have had a fever for the past 3 days. You have a stiff neck. Your eyes are sensitive to light and a normal amount of light makes you squint your eyes and your head hurt even more.

Deep vein thrombosis – Imagine your right leg has been hurting and swollen for the past 5 days. Your right leg is also more sensitive and redder compared to your left leg. When you touch the back of your knee it hurts even more. You have previously visited the doctor due to your high blood pressure and you have had complications with your heart in the past. Lately you have been laying down a lot while recovering from pneumonia.

Pneumonia – Imagine you have had a wet cough and a fever for the past 3 days. You have a fever of 38.3°C. You also have a high blood pressure and shortness of breath making it difficult for you to breath so you have to breath quite fast. You also feel more tired than normal.

*Requires non-emergent care*

Influenza – Imagine you have felt quite weak, and you have had a fever for the past 2 days. Now you have a fever of 38.1°C, your head hurts, and you have a cough. The symptoms appeared suddenly. Your coworkers have been sick lately. You have not received the influenza vaccine yet.

Back pain – Imagine you were shoveling snow 3 weeks ago and your lower back has hurt since. You find it difficult to raise your right foot when you walk. Your toes are also a bit numb.

*Self-care appropriate*

Acute bronchitis – Imagine you have had a cough for the past 12 days. You feel like there is a lot of sputum in your throat and your throat is a bit sore. You have not had a fever. Lately you have not met anyone sick with similar symptoms.
