# Supplementary material for: Testing Two Online Symptom Checkers With Vulnerable Groups: Usability Study to Improve Cognitive Accessibility of eHealth Services
Source: JMIR Hum Factors. 2024 Mar 8;11:e45275. doi: 10.2196/45275 (PMC10960212; doi:10.2196/45275)
Supplement: Multimedia Appendix 3 [file humanfactors_v11i1e45275_app3.docx]

Multimedia Appendix 3

The background information of the participants. MID stands for mildly intellectually disabled. All the participants used digital services multiple times a day.

| Group | Gender | Age | Number of visits to the doctor in the past 2 years | Number of diagnosed medical conditions | Country of origin (region) |
| --- | --- | --- | --- | --- | --- |
|  |  |  |  |  |  |
| Older adults | Male | 75–79 | 0–5 | 2–3 | Finland |
| Older adults | Female | 75–79 | 0–5 | 2–3 | Finland |
| Older adults | Male | 75–79 | 0–5 | 0–1 | Finland |
| Older adults | Female | 75–79 | 10+ | 4–5 | Finland |
| MID | Male | 25–29 | 0–5 | 0–1 | Finland |
| MID | Male | 35–39 | 0–5 | 0–1 | Finland |
| MID | Female | 20–24 | 0–5 | 2–3 | Finland |
| MID | Female | 30–34 | 0–5 | 0–1 | Finland |
| Non-native | Female | 30–34 | 0–5 | 2–3 | Central-West Africa |
| Non-native | Male | 40–44 | 0–5 | 0–1 | East or South Europe |
| Non-native | Female | 20–24 | 0–5 | 0–1 | East or South Europe |
| Non-native | Female | 40–44 | 10+ | 2–3 | East or South Europe |
| Non-native | Female | 50–54 | 6–9 | 2–3 | Middle East |
