# Supplementary material for: Testing Two Online Symptom Checkers With Vulnerable Groups: Usability Study to Improve Cognitive Accessibility of eHealth Services
Source: JMIR Hum Factors. 2024 Mar 8;11:e45275. doi: 10.2196/45275 (PMC10960212; doi:10.2196/45275)
Supplement: Multimedia Appendix 4 [file humanfactors_v11i1e45275_app4.docx]

## **Multimedia Appendix 3 – Usability Problem Categories**

A summary of the discovered usability problems including how many individual problems were grouped under the category and how many times the users in total encountered the problems within the category.

| Usability Problem Category | Number of Individual Problems | Number of Users Who Encountered the Problems |
| --- | --- | --- |
|  |  |  |
| Clarity of answering options | 13 | 39 |
| Visibility of information | 9 | 30 |
| Intuitive and consistent interactions | 7 | 33 |
| Long, uncommon, and compound words | 5 | 11 |
| Input methods | 4 | 13 |
| Clarity or visibility of instructions | 4 | 11 |
| Guiding the user’s focus | 4 | 10 |
| Consistency of external links (the pages that were opened through the external links were different by their appearance and logic) | 4 | 5 |
| Icons, symbols, and abbreviations | 3 | 6 |
| Navigation | 3 | 6 |
| Informing the user of errors | 3 | 5 |
| Allowing the user to revert their actions | 3 | 3 |
| Allowing the user to express themselves freely | 2 | 16 |
| Responsiveness or technical issue (the UI did not react to user actions as expected) | 2 | 2 |
