# Supplementary material for: Testing Two Online Symptom Checkers With Vulnerable Groups: Usability Study to Improve Cognitive Accessibility of eHealth Services
Source: JMIR Hum Factors. 2024 Mar 8;11:e45275. doi: 10.2196/45275 (PMC10960212; doi:10.2196/45275)
Supplement: Multimedia Appendix 5 [file humanfactors_v11i1e45275_app5.docx]

Multimedia Appendix 5

Average SUS scores for both services. For older adults the two services got the same results, for the two other groups Service A got a slightly better score.

| Average SUS scores | Service A | Service B |
| --- | --- | --- |
|  |  |  |
| Older adults | 70.6 | 70.6 |
| MID | 73.8 | 65 |
| Non-natives | 81.5 | 78.5 |
